# Experimentally Testing the Hypothesis That Hybrid Fitness Can Increase Evolutionarily: An Example in Spadefoot Toads

**DOI:** 10.1002/ece3.72531

**Published:** 2025-11-27

**Authors:** Sedona Ryan, Patrick Kelly, Bryson Loflin, Karin S. Pfennig

**Affiliations:** ^1^ Environment, Ecology and Energy Program University of North Carolina at Chapel Hill Chapel Hill North Carolina USA; ^2^ Department of Biology University of North Carolina at Chapel Hill Chapel Hill North Carolina USA

**Keywords:** adaptive hybridization, Bateson‐Dobzhansky‐Muller, genetic incompatibilities, introgression, reinforcement, speciation

## Abstract

Hybrids often have reduced fitness because of genetic incompatibilities. If populations contain variation at loci involved in these genetic incompatibilities, then selection can disfavor alleles involved in incompatibilities, thereby reducing their frequency within the parent‐lineages' gene pools in the hybridizing population. Such selection can concomitantly increase hybrid fitness in populations over evolutionary time. This hypothesis predicts that hybrid fitness should be higher in populations where species have hybridized for longer periods (or where hybridization is more frequent) versus in populations where species have hybridized for a shorter duration (or where hybridization is less frequent). We tested this prediction in spadefoot toads, *Spea bombifrons* and *Spea multiplicata* . *Spea bombifrons* has expanded its range into that of *S. multiplicata* ; consequently, regions of sympatry differ in the length of time during which hybridization has occurred. We used a “space for time substitution” experiment to contrast hybrid fitness, as measured by development time and body size, in different regions of sympatry. We found that hybrids derived from “Old sympatry” were larger and had faster development than hybrids derived from “New sympatry.” Despite limits in our study design, our results suggest that selection can reduce incompatibilities and increase hybrid fitness evolutionarily. We discuss means for best evaluating whether selection can evolutionarily reduce genetic incompatibilities. Doing so is important to better understand ecological and evolutionary processes ranging from speciation to hybridization's role in adaptive evolution and species range expansion.

## Graphical abstract

Low hybrid fitness is often generated by “genetic incompatibilities” that arise between the parental species' genomes. Our study provides experimental evidence that selection against such incompatibilities might result in the evolutionary increase of hybrid fitness over time. These results have important implications for the evolution and maintenance of reproductive isolation between species.

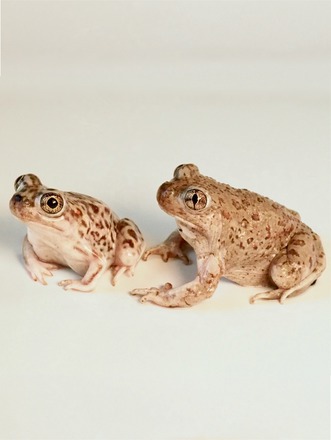

## 1 Introduction

Hybridization—interbreeding between species—is often considered deleterious; hybrids can have reduced fertility or survival relative to parental types. Such deleterious effects are generally driven by genetic incompatibilities between the parent species that are revealed in hybrids (Coyne and Orr 2004; Seehausen et al. 2014; Mack and Nachman 2017). Hybrid fitness therefore provides a window into genetic incompatibilities, especially when hybrid fitness is a product of intrinsic, developmental factors as opposed to extrinsic, ecological factors (Coyne and Orr 2004).

The fitness of hybrids relative to parental types can vary within a population, especially if parental types within a population are genetically variable such that allelic combinations are differentially incompatible in hybrid offspring (for reviews and examples see Reed and Markow 2004; Cutter 2012; Matute et al. 2014; Larson et al. 2018; Coughlan and Matute 2020; Kalirad et al. 2024). If such variation exists, selection within populations can reduce the incidence and severity of incompatibilities by disfavoring alleles in the parent lineages' gene pools that contribute to lower hybrid fitness and/or by favoring alleles that mitigate incompatibilities (Barton and Hewitt 1985; Sanderson 1989; Virdee and Hewitt 1994; Schilthuizen et al. 1999, 2004; Seehausen 2004; Schilthuizen and Lammers 2013; Seidl et al. 2019). Consequently, relative hybrid fitness within a given population would improve over evolutionary time.

To date, most work on intrinsic incompatibilities has focused on their accumulation between taxa (e.g., Matute et al. 2010; Moyle and Nakazato 2010; Schilthuizen and Lammers 2013; Wang et al. 2015; Kalirad et al. 2024). Consequently, the conditions under which hybrid fitness might evolutionarily increase owing to an evolutionary *reduction* in incompatibilities remain unclear. One expectation is that, for selection to favor reduced incompatibilities, hybridization must be ongoing and sufficiently common. If hybrids are rarely produced or are produced in a “pulse” event, then selection disfavoring incompatible alleles (or favoring alleles that mitigate incompatibilities) within the population would likely be relatively weak.

A more problematic issue is that, when hybridization incurs fitness costs, selection's efficacy infavoring traits in the parent species that eliminate hybridization could be stronger than selection's efficacy in removing incompatibilities between them. Indeed, if hybridization is common, the evolution of traits that prevent hybridization (i.e., reinforcement; see reviews in Servedio and Noor 2003; Coyne and Orr 2004; Price 2008; Pfennig and Pfennig 2012) could arise more readily—and more rapidly—than either the loss of alleles in populations that contribute to low hybrid fitness or the gain of alleles that mitigate incompatibilities. In short, repeated hybridization generates a situation in which incompatibilities are more readily exposed to selective removal (Pfennig 2021), but the expression of incompatibilities generates selection favoring the evolution of traits that reduce hybridization (Servedio and Noor 2003; Coyne and Orr 2004; Price 2008; Pfennig and Pfennig 2012). As traits evolve that reduce hybridization, selection disfavoring incompatibilities is weakened because hybrids are no longer being produced. Thus, the extent to which selection removes incompatibilities might derive from a balance between the evolution of traits that reduce hybridization versus the efficacy with which selection can eliminate the alleles involved in genetic incompatibilities (or favor the spread of alleles that mitigate those incompatibilities).

The possibility that selection might disfavor incompatibilities, enhance hybrid fitness, and effectively reduce postzygotic isolation over time needs testing (Schilthuizen and Lammers 2013). Doing so would clarify the factors that govern the extent and dynamics of gene flow between species. Increasing evidence suggests that gene flow between species, especially “adaptive introgression,” is important in ecological and evolutionary processes (Hedrick 2013; Pfennig et al. 2016; Taylor and Larson 2019; Edelman and Mallet 2021; Pfennig 2021). Indeed, hybridization with introgression can enable species to respond to global change by facilitating population rescue, rapid adaptation, and/or movement into new habitats (Song et al. 2011; Hedrick 2013; Taylor et al. 2015; Pierce et al. 2017; Oziolor et al. 2019; DeVos et al. 2023). Adaptive introgression can occur even with low hybrid fitness (Abbott et al. 2013; Edelman and Mallet 2021; Pfennig 2021). Nevertheless, selective removal of alleles involved in incompatibilities (or selective favoring of alleles that mitigate incompatibilities) in populations where hybridization is occurring could reduce population genetic load while enhancing the likelihood that hybridization promotes adaptive evolution, population persistence, or expansion of populations into new niches (Schilthuizen et al. 2004; Seehausen 2004; Schilthuizen and Lammers 2013).

If selection mitigates genetic incompatibilities evolutionarily, then hybridization frequency or hybridization duration should predict hybrid fitness. Specifically, hybrids should have higher fitness in populations with relatively more frequent exposure of hybrids to selection or a longer period during which hybrids have been exposed to selection. Testing the prediction that hybridization frequency predicts hybrid fitness requires measuring hybrid fitness across populations with divergent rates of hybridization; however, population‐specific hybridization rates (and the degree to which they vary over time) are not always known. Alternatively, if populations are known (or can be inferred) to differ in the length of time over which they have been sympatric and hybridizing, it becomes possible to assay whether hybrid fitness is higher in populations where the hybridizing species have been in contact for longer.

Systems in which range expansions have occurred and are accompanied by hybridization (Pfennig et al. 2016) offer cases in which both the length of time in sympatry varies and the *relative* length of time populations have been sympatric can be inferred. Specifically, if one species expands its range into that of another species, and these two species hybridize when in contact, then populations at the range's leading edge will necessarily constitute more recent sympatry than populations behind the range edge. Such situations provide the opportunity to use “space for time substitution” experiments (Wogan and Wang 2018; Lovell et al. 2023) to contrast hybrid fitness in newer sympatry at the range edge with hybrid fitness in relatively older sympatry behind the range edge. Although space for time substitution experiments leverage geographic variation in populations to make inferences about temporal dynamics in ecology or evolution (Wogan and Wang 2018), they do not control for local ecological, demographic, or population genetic factors (Lovell et al. 2023). Nevertheless, they can provide a first step in examining evolutionary hypotheses that are otherwise difficult to test directly—as is the case with predictions about the evolutionary loss of alleles involved in hybrid incompatibilities and/or the evolutionary increase in hybrid fitness (see also Seidl et al. 2019).

Here, our goal is to provide an example of a space for time substitution experiment to examine the evolution of hybrid fitness and the potential for selection to remove alleles involved in hybrid incompatibilities. In highlighting how such an approach can be used, we discuss its limitations and suggest ways by which future studies might provide more conclusive insights.

For our study, we used naturally hybridizing spadefoot toads, *Spea bombifrons* and *Spea multiplicata* from the western USA. Hybridization between these two *Spea* species carries major fitness costs derived from genetic incompatibilities: hybrid males are sterile, and hybrid females are partially fecund (Simovich et al. 1991; Wünsch and Pfennig 2013). Because of these fitness costs, selection has favored the evolution of mating behaviors, including mate choice, that minimize hybridization (i.e., reinforcement has occurred; Pfennig 2000, 2003, 2007; Pfennig and Pfennig 2005; Pfennig and Stewart 2011; Pfennig and Rice 2014; Calabrese and Pfennig 2022).

Yet, hybridization can be fitness enhancing for *S. bombifrons* females (Pfennig and Simovich 2002; Pfennig 2007; Chen and Pfennig 2020a, 2020b). Spadefoots breed in highly ephemeral pools that often dry before tadpoles can metamorphose (Pfennig and Simovich 2002). Hybrids develop significantly faster than *S. bombifrons* tadpoles, so they are more likely to escape a drying pond (and therefore survive) than *S. bombifrons* tadpoles (Pfennig and Simovich 2002; Pfennig 2007). Consequently, female *S. bombifrons* assess pond depth (a proxy of pond longevity) and preferentially hybridize with *S. multiplicata* males in shallow ponds (Pfennig 2007; Chen and Pfennig 2020a; see also Chen et al. 2022). Moreover, females do not simply mate randomly with *S. multiplicata* males: they prefer heterospecific males that produce hybrid offspring with higher fitness (Chen and Pfennig 2020a, 2020b).

The evolution of this facultative hybridization potentially contributed to the ability of *S. bombifrons* to expand its range into the desert southwestern USA (Pierce et al. 2017). Prior work suggests that *S. bombifrons* has expanded out of its ancestral grassland habitat (which is more mesic) into xeric habitat that is more like *S. multiplicata* 's ancestral habitat (Rice and Pfennig 2008; Chunco et al. 2012; Pierce et al. 2017). Introgression from *S. multiplicata* is associated with this expansion by *S. bombifrons* into the southwestern USA (Pierce et al. 2017).

Because of these dynamics, hybridization is ongoing despite reinforcement via the evolution of mating behaviors that reduce hybridization (Pfennig 2000, 2003, 2007; Pfennig and Pfennig 2005; Pfennig and Stewart 2011; Pfennig and Rice 2014; Seidl et al. 2019; Calabrese and Pfennig 2022; Chen et al. 2022). Given that hybridization continues to occur (Simovich and Sassaman 1986; Pfennig and Simovich 2002; Pfennig et al. 2012), natural selection could reduce incompatibilities in sympatric populations (Seidl et al. 2019). Moreover, because *S. bombifrons* has expanded its range southwestward (Rice and Pfennig 2008; Pierce et al. 2017), populations in sympatry vary in the duration that they have been in contact with *S. multiplicata* . More northeastern populations of *S. multiplicata* in the region of Texas have been in contact with *S. bombifrons* for longer (and therefore have presumably been experiencing hybridization for longer) than more southwestern populations in Arizona. This spatial variation can therefore serve as a proxy for the length of time during which selection could act to reduce incompatibilities (i.e., “space” can serve as a proxy for “time”; sensu Wogan and Wang 2018; Lovell et al. 2023).

Prior work using gene expression to study incompatibilities in *Spea* found evidence consistent with the possibility that selection has reduced incompatibilities across sympatry (Seidl et al. 2019). However, this previous work did not measure hybrid fitness. Here, we examine whether hybrid fitness, as measured by tadpole growth and survival over a 12‐day period, varies across different populations as expected if selection has reduced genetic incompatibilities over time. We specifically examined whether hybrid fitness was higher in populations that have been in contact longer, as is expected under the hypothesis that selection can evolutionarily reduce genetic incompatibilities between species.

## 2 Materials and Methods

We created two treatments, a northern “Old sympatry” treatment and southern “New sympatry” treatment, by breeding allopatric *S. bombifrons* females from their ancestral habitat with *S. multiplicata* males from Texas (“Old sympatry”) and Arizona (“New sympatry”). We varied only the region type of the male for two key reasons. First, the environment can create differences in female condition that generate maternal effects that can confound studies of genetic incompatibilities. Such effects can be evaluated (and controlled) to some extent by a fully factorial design in which the male and female region of origin are varied. However, this fully factorial design requires many more animals than we had available and, more critically, it cannot disentangle maternal effects per se (e.g., differential egg investment in grassland versus desert environments) from mitonuclear incompatibilities in hybrids that also can vary across populations. Given these potential confounding effects, controlling the female region was imperative.

Second, previous work has shown that male *S. multiplicata* vary in the fitness (in terms of size and development rate) of hybrid offspring they sire, male call predicts this variation, and *S. bombifrons* females prefer *S. multiplicata* males that produce hybrid tadpoles with higher fitness (Chen and Pfennig 2020a, 2020b). This variation in fitness of hybrids sired by different males indicates that variation in incompatibilities exists and can be exposed to selection. Indeed, mate choice by *S. bombifrons* could facilitate this process.

Given these factors, we opted to use allopatric females as a consistent background against which to test the effect of region by pairing them with males that differed in their region of origin. We provide details of the nearest town of collection in Table 1 for each of the families that were used in the experiment (see Methods below for details on breeding and tadpole rearing). For these sites, the males in Texas were all collected within 30 miles of one another; the males from Arizona were all collected from within 2 miles of one another. The animals from Texas were therefore more broadly distributed than those from Arizona. Ideally, we would have sampled more extensively across both regions (see Section 4).

**TABLE 1:** Size and collection details for adults that were bred to create tadpoles reared in our growth experiment. Family ID corresponds to family IDs used throughout the paper.

Family ID	Dam locale	Dam year collected	Dam SVL (mm)	Dam mass (g)	Sire sympatry (treatment)	Sire locale	Sire year collected	Sire SVL (mm)	Sire mass (g)
1	Garden State, KS	2019	50.39	13.39	New	Rodeo, NM	2019	49.69	12.29
7	Calhoun‐Byers, CO	2019	52.01	12.22	New	Rodeo, NM	2017	43.00	10.36
9	Syracuse, KS	2023	59.85	18.84	New	Rodeo, NM	2019	44.96	8.9
10	Syracuse, KS	2023	59.85	17.57	Old	Plainview, TX	2023	43.82	6.68
12	Syracuse, KS	2023	56.2	15.97	Old	Hale Center, TX	2023	40.11	7.68
14	Rolla, KS	2023	48.44	13.76	Old	Abernathy, TX	2023	43.02	8.07

Animals were collected from natural populations as reproductively mature adults and returned to lab facilities at UNC. There, they were maintained a minimum of 5 months to a maximum of 6 years prior to this experiment (spadefoots can live over 10 years). Although the use of animals that varied in their time in the lab is not ideal, the unpredictability of spadefoot breeding combined with the lingering impacts of COVID on our research dictated an opportunistic use of animals that were available. Note that the distribution of ages in the lab was such that males with longer periods in the lab were used for the New sympatry treatment (Table 1). Previous work has shown that males that have been in the lab longer produce tadpoles with better growth (Harmon et al. 2023). Thus, regarding time in the lab, our design was conservative because we predicted that the New sympatry treatment should have reduced growth and survival compared to the Old sympatry treatment.

We measured all adults for mass and body size (i.e., snout‐to‐vent length, SVL) at the start of the experiment. To induce breeding, we injected adults with 0.07 mL of 0.01 μg/μL luteinizing hormone releasing hormone solution and placed each pair separately in a tank filled with dechlorinated water. Four families from each treatment produced hybrid egg clutches. However, two families (one from each treatment) had almost total tadpole mortality so that no offspring from these families could be reared in the growth experiment. Because this mortality occurred in one family per treatment, it did not bias our findings. Thus, we reared tadpoles from three pairs from each treatment (i.e., six total families; Table 1). On Day 12, we reared 80 tadpoles per family individually in small water‐filled tanks (18 cm × 13 cm × 8.5 cm) interspersed in the rearing room with their location recorded.

Despite being reared in a controlled common lab environment, differential growth might arise if hybrids from the different sympatric regions possess behavioral differences in foraging that impact growth (Pfennig and Murphy 2000; Pfennig et al. 2007). We therefore assayed tadpole behavior to evaluate whether growth differences between our treatments, if any, might reflect extrinsic (i.e., ecological) effects as opposed to intrinsic (i.e., genetic/developmental) effects. Specifically, we video recorded each tadpole in its rearing container for 5 min. The videos were analyzed using Noldus Ethovision XT software, which automatically detected the tadpole and measured total activity time (i.e., the total time of tadpole movement during the recording). Ethovision is an automatic tracking system, so behavioral measurements were taken without knowledge of treatment.

On Day 24, tadpoles were euthanized by immersion in MS 222 and preserved in 95% ethanol (*N* = 422). *Spea* can reach metamorphosis in as little as 3 weeks, so 24‐day‐old tadpoles are far enough along in development to exhibit fitness differences if any. Following preservation, snout‐to‐vent length (SVL, mm), mass (g), and Gosner stage (GS, a measure of development; Gosner 1960) were recorded for each tadpole. These are good fitness proxies, because development rate determines survival in the rapidly drying ponds in which the tadpoles develop (Pfennig and Simovich 2002; Pfennig 2007), and size can impact subsequent survival and fitness (Pfennig and Pfennig 2005; but also see de la Serna Buzon et al. 2020).

We performed all analyses in R version 4.4.0 (R Core Team 2024). We used packages lme4 (Bates 2014) and lmerTest (Kuznetsova et al. 2017) for mixed‐effects modeling and inference and packages effects (Fox 2003; Fox and Weisberg 2018a, 2018b) and parameters (Lüdecke et al. 2020) for extracting model estimates. We used Kenward‐Roger approximations to degrees of freedom for mixed model inference and post hoc tests. We used package ggplot2 for visualization (Wickham 2016).

To check for family‐level differences in mortality, we initially specified a generalized linear mixed model with a binomial response, family as a fixed effect, and random intercepts for family and rearing location to account for nonindependence. However, both random effects had standard deviations of zero in this model, so to avoid singularity, we instead report findings from a generalized linear model (no random effects) with a binomial response (results did not differ between models).

**FIGURE 1:**
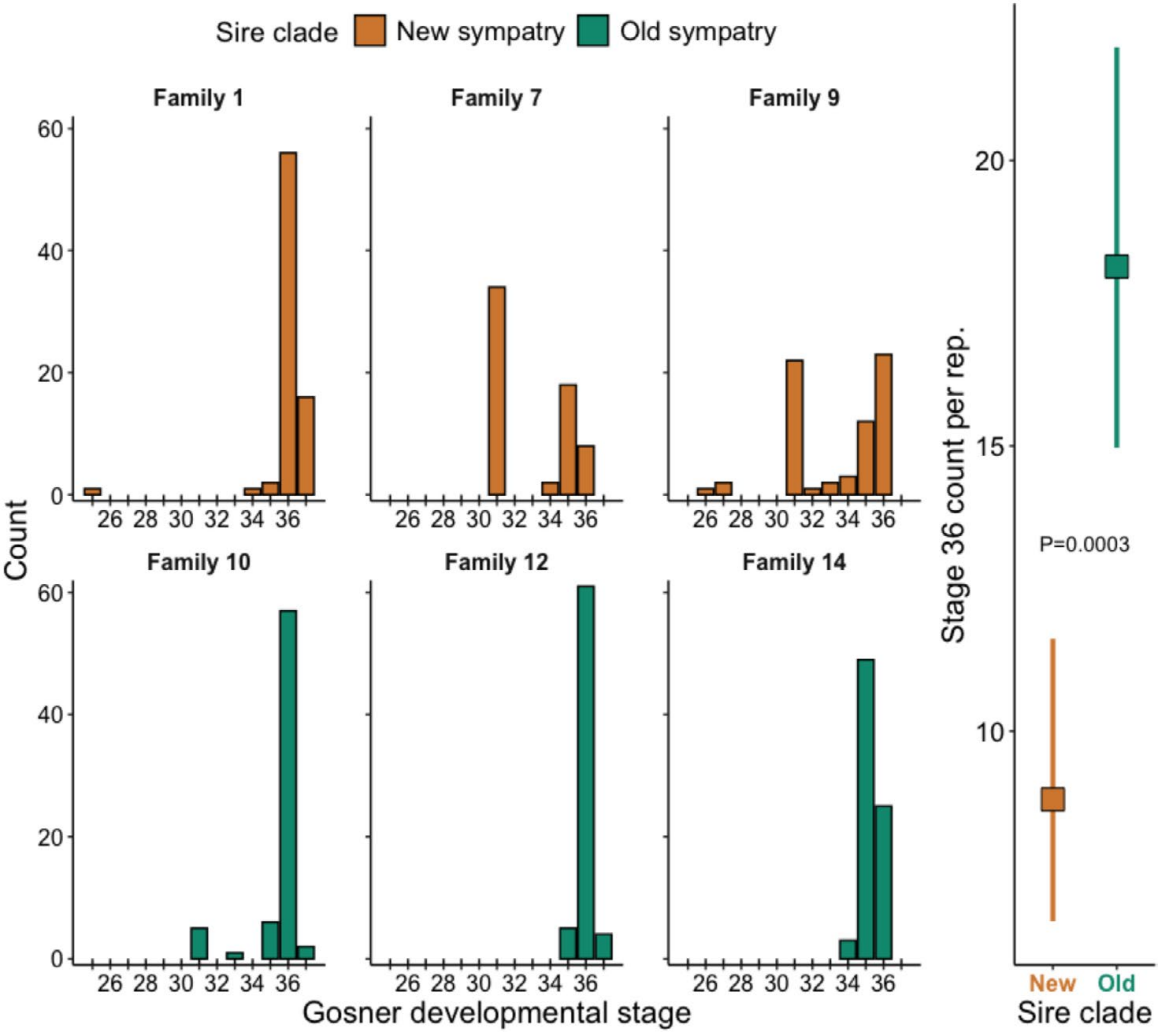
Left subpanels: Counts of tadpoles that achieved each Gosner developmental stage by family. Right panel: Mean count and 95% confidence intervals for each treatment showing that, on average per family and per rearing location, more Old sympatry tadpoles achieved stage 36 (the highest stage with all six families represented and the median stage for both treatments; see Section 2) than New sympatry tadpoles, indicating that Old sympatry tadpoles developed faster.

To compare treatment development rates, we analyzed the number of tadpoles achieving Gosner developmental stage (GS) 36, the highest stage with all six families represented and the median stage for both treatments (see Section 3 and Figure 1). We used a generalized linear model with Poisson distribution to model this as the rate at which each treatment's tadpoles developed to GS 36 per family‐rearing location replicate. We calculated body condition as the standardized mass index (SMI) (Peig and Green 2009; MacCracken and Stebbings 2012). SMI uses the slope coefficient from standardized major axis regression of ln mass on ln body length (in this case, SVL) as the scaling exponent in calculating weight‐for‐body length (SMI).

We applied Box–Cox power transformations to tadpole SVL and mass data prior to analysis in order to correct for left skew; we used the R package MASS (Venables and Ripley 2002) to separately calculate transformation values for each variable. We log‐transformed tadpole SMI data and square‐root transformed tadpole movement data prior to analysis. We back transformed all variables for visualization.

**FIGURE 2:**
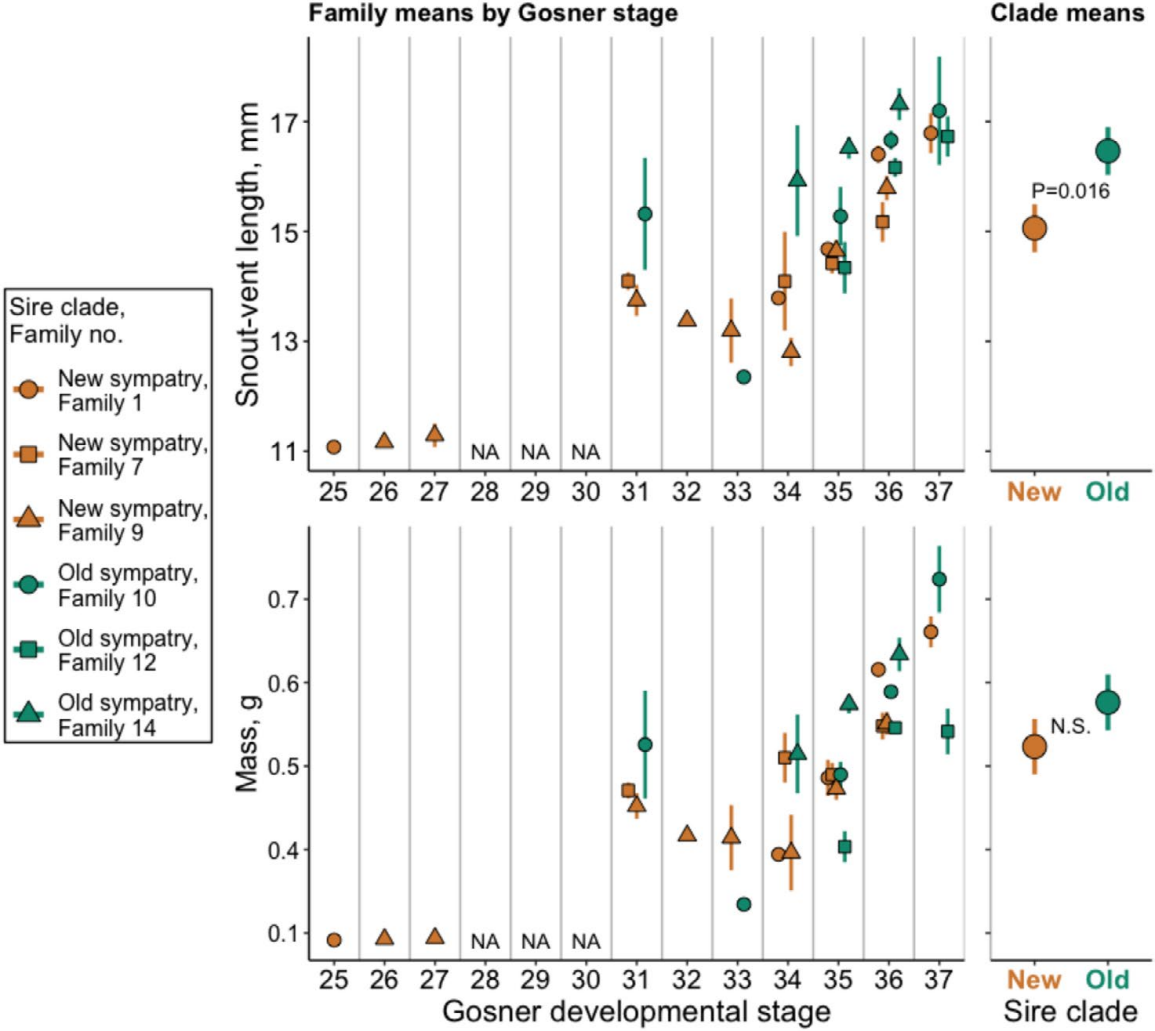
Left panels: Family unadjusted mean (SE) of mass and SVL by Gosner developmental stage for New sympatry (brown) and Old sympatry (green). Right panel: Points with error bars are treatment means and 95% confidence intervals from a generalized linear model that includes Gosner stage and sire SVL as predictor variables. Generally, tadpoles increase with size over development. Thus, Old sympatry tadpoles were larger in SVL than New sympatry even when accounting for their faster development.

We analyzed tadpole SVL, mass, SMI, and movement data as responses in separate linear mixed‐effects models with treatment, sire SVL, and tadpole GS as fixed effects. We included sire SVL as a covariate because prior studies have demonstrated associations between sire body size and offspring phenotypes in *Spea* (Pfennig 2008; Kelly et al. 2019, 2021; Chen and Pfennig 2020a). We included GS to determine whether any treatment differences in tadpole size, SMI, or movement were attributable to observed treatment differences in development rate (see Section 3). In each model except the one with movement data, we included random intercepts for family and rearing location to account for nonindependence. To avoid singularity, the movement model included only family as a random effect, as rearing location had a standard deviation of 0 in this model. For GS, SVL, and mass models, we produced marginal‐effects plots for the effect of treatment (Figures 1 and 2).

## 3 Results

We found no evidence of differential survival among experimental families (Table 2).

**TABLE 2:** Results from binomial regression evaluating mortality for Old versus New sympatry. No evidence of differential survival was detected. Family 1 (New sympatry treatment) was the baseline. Confidence intervals of “NA” for Families 12 and 14 reflect the fact that those families had no mortality, which precluded estimation of profile‐likelihood upper bounds. The equal‐tailed confidence intervals for these families are [−4137.29, 4169.76] and [−3944.00, 3976.47] respectively.

Parameter	Treatment	Log‐odds	SE	95% CI	*z*	*p*
(Intercept)		4.33	1.01	[2.83, 7.20]	4.3	1.69E‐05
Family 7	New	−1.3	1.17	[−4.32, 0.78]	−1.12	0.2646
Family 9	New	−1.53	1.13	[−4.51, 0.41]	−1.35	0.1767
Family 10	Old	−0.07	1.42	[−3.31, 3.17]	−0.05	0.9619
Family 12	Old	16.24	2119.18	[−163.90, NA]	7.66E‐03	0.9939
Family 14	Old	16.24	2020.56	[−155.52, NA]	8.04E‐03	0.9936

However, we did find that tadpoles from Old sympatry developed faster than those from New sympatry. Specifically, tadpoles from both treatments achieved median Gosner developmental stages (GS) of 36, which was also the highest GS at which all six experimental families were represented (Figure 1). However, a total of 143 Old sympatry tadpoles achieved GS 36, compared to 87 New sympatry tadpoles. This pattern was reflected in our model results: Old sympatry tadpoles developed to GS 36 at an average rate of 18.1 ± 1.8 (1 SE) tadpoles per family‐rearing location replicate, whereas New sympatry tadpoles' average rate was 8.8 ± 1.3 (Table 3).

**TABLE 3:** Results from Poisson regression, showing more‐rapid development of Old sympatry tadpoles. Old sympatry was the baseline.

Parameter	Log‐mean	SE	95% CI	*z*	*p*
(Intercept)	−1.04	0.15	[−1.34, −0.75]	−6.93	4.13E‐12
Sire SVL	0.25	0.09	[0.07, 0.44]	2.66	0.0077
Clade [New sympatry]	0.72	0.2	[0.34, 1.12]	3.66	0.0003

Old sympatry tadpoles also grew larger in terms of snout‐vent length (SVL; Table 4), though we found no difference between the treatments in mass (Table 5) or body condition (SMI; Table 6). Faster development (higher GS) accounted for treatment differences in mass (Table 5) and SVL as is expected because tadpoles are larger at later stages. Nevertheless, the treatment difference in SVL was significant in addition to GS (Table 4). In other words, Old sympatry tadpoles were larger in SVL than New sympatry tadpoles even when accounting for faster development.

**TABLE 4:** Results from linear mixed‐effects model contrasting snout‐vent length (SVL) of tadpoles from Old and New sympatry treatments. Old sympatry was the baseline.

Parameter	Coefficient	SE	95% CI	*t*	df	*p*
**Fixed effects**						
(Intercept)	−18.46	12.21	[−19.66, −16.91]	−6.16	283.93	2.45E‐09
Sire SVL	9.56	7.54	[−5.35, 11.29]	2.84	3.23	0.0604
Clade [New sympatry]	12.27	8.84	[9.45, 13.81]	4.24	3.62	0.0164
Gosner stage	8.99	5.48	[8.48, 9.41]	8.81	355.02	5.77E‐17
**Random effects**						
SD (Intercept: Family)	8.35	7.33	[6.50, 10.73]			
SD (Intercept: Rearing location)	7.3	6.92	[5.13, 10.39]			
SD (Residual)	12.04	5.61	[11.85, 12.23]			

**TABLE 5:** Results from linear mixed‐effects model contrasting mass of tadpoles from Old and New sympatry treatments. Old sympatry was the baseline.

Parameter	Coefficient	SE	95% CI	*t*	df	*p*
**Fixed effects**						
(Intercept)	−0.76	1.04	[−0.86, −0.65]	−18.12	280.82	3.82E‐49
Sire SVL	1.02	1.01	[1.00, 1.04]	2.83	3.12	0.0633
Clade [New sympatry]	1.03	1.01	[0.99, 1.06]	2.09	3.33	0.1188
Gosner stage	1.01	1	[1.01, 1.01]	9.2	404.87	1.86E‐18
**Random effects**						
SD (Intercept: Family)	1.01	1.01	[1.00, 1.03]			
SD (Intercept: Rearing location)	1	1	[1.00, 1.02]			
SD (Residual)	1.04	1	[1.03, 1.04]			

**TABLE 6:** Results from linear mixed‐effects model contrasting scaled mass index (SMI) of tadpoles from Old and New sympatry treatments. Old sympatry was the baseline.

Parameter	Coefficient	SE	95% CI	*t*	df	*p*
**Fixed effects**						
(Intercept)	1.01	1.18	[0.73, 1.41]	0.07	201.9	9.43E‐01
Sire SVL	1	1.01	[0.96, 1.05]	0.31	3.42	0.7761
Clade [New sympatry]	0.93	1.03	[0.86, 1.01]	−2.27	4.12	0.0841
Gosner stage	1.01	1.01	[1.00, 1.02]	1.28	228.41	2.01E‐01
**Random effects**						
SD (Intercept: Family)	1.02	1.02	[1.00, 1.11]			
SD (Intercept: Rearing location)	1.01	1.01	[1.00, 1.09]			
SD (Residual)	1.17	1.01	[1.16, 1.19]			

Differences in activity could account for our treatment differences if they differ in foraging. However, the Old and New sympatry tadpoles did not differ significantly in their movement (Table 7).

**TABLE 7:** Results from linear mixed‐effects model contrasting linear movement of tadpoles from Old and New sympatry treatments. Old sympatry was the baseline.

Parameter	Coefficient	SE	95% CI	*t*	df	*p*
**Fixed effects**						
(Intercept)	15563.06	499.63	[6512.58, 28494.60]	5.58	220.92	6.96E‐08
Sire SVL	17.33	22.19	[359.77, 113.26]	−0.88	3.07	0.4406
Clade [New sympatry]	10.01	85.63	[1003.15, 642.39]	−0.34	3.18	0.7538
Gosner stage	2.03	0.41	[7.22, 0.03]	−2.22	405.41	2.71E‐02
**Random effects**						
SD (Intercept: Family)	71.04	14.04	[12.44, 405.69]			
SD (Residual)	406.05	0.5	[353.74, 466.09]			

## 4 Discussion

We found that hybrid tadpoles derived from populations where hybridization has presumably been occurring for a longer time (“Old sympatry”) grew better in terms of development rate and body size than those from populations where hybridization has presumably been occurring for a shorter time (“New sympatry”). These results are consistent with the hypothesis that ongoing exposure of hybrids to natural selection can reduce genetic incompatibilities over time and thereby increase hybrid fitness evolutionarily (Barton and Hewitt 1985; Sanderson 1989; Virdee and Hewitt 1994; Schilthuizen et al. 1999, 2004; Seehausen 2004; Schilthuizen and Lammers 2013; Seidl et al. 2019).

However, several shortcomings in our experiment limit our ability to make strong conclusions from these data. First, the extremely small number of families for each treatment type means that our results should be generalized with caution. We necessarily lacked representative sampling of populations across the different regions, and it is therefore unclear whether these families adequately represent their populations and regions.

Moreover, our design could not account for population variation in rates of hybridization that would impact the extent to which selection has removed alleles involved in incompatibilities. Previous work has shown that hybridization rates can vary within and across populations (Pfennig and Simovich 2002; Pfennig 2003; Pfennig et al. 2012; Pierce et al. 2017). We lacked information about historical rates of hybridization for some of the populations used (specifically in “Old sympatry”), so we could not evaluate how population‐specific hybridization rates affect the evolution of incompatibilities. Indeed, this was a key reason we focused on comparing regions (see Section 1 for rationale of comparing “Old” and “New” sympatry). Generally, populations with higher rates of hybridization should experience increased efficacy in the selective removal of alleles involved in incompatibilities (see Section 1). An alternative approach to testing the hypothesis that selection removes alleles involved in incompatibilities (or favors alleles that mitigate incompatibilities) is to evaluate whether a positive relationship exists between hybrid fitness and hybridization rate across populations.

Generally, we would expect that variation across populations in hybridization rate might diminish—rather than exaggerate—differences between regions that differ in the time over which hybridization has occurred. In our study for example, if hybridization rate is higher in New sympatry, then this would tend to diminish differences from Old sympatry where hybridization has been ongoing for longer periods. So, we suggest that the possibility that populations vary in hybridization within region in a way we failed to sample does not undercut the value of our results. Nevertheless, future studies are needed that consider both local and regional factors in evaluating whether alleles involved in incompatibilities can be selectively removed over time.

A second shortcoming of our study is that our measure of fitness was restricted to survival and growth in the tadpole stage. Although the tadpole stage in *Spea* is a period of strong selection, incompatibilities in hybrids are manifest in other components of fitness such as fertility, survival into adulthood, or reproductive success (Simovich and Sassaman 1986; Pfennig and Simovich 2002; Schmidt and Pfennig 2016; Kelly 2020). For example, hybrids in *Spea* show reduced fertility (Simovich 1985; Wünsch and Pfennig 2013), so alleles that contribute to incompatibilities involved in reduced fertility should be strongly disfavored by selection. Additional studies are needed to determine if these other fitness components show patterns consistent with the possibility that selection has ameliorated hybrid incompatibilities. Indeed, our finding of no differences in survival could be interpreted to suggest that the strongest incompatibilities have not shown any differential evolution between Old and New sympatry (although it could be attributed to low power in our design). That our sample sizes in both treatments were equally diminished by families in which nearly all tadpoles failed to develop could reflect equally deleterious incompatibilities in both population types. Re‐pairing these families to establish if they again fail and identifying the genotypes involved in hybrid survival are needed to better evaluate these possibilities.

Related to the above issues, a further limitation of our study is that we restricted our contrasts to hybrids. Because we did not include parental types, we do not know how hybrids in our different regions would have performed relative to parentals within their own populations (or to parentals from outside sympatry). If alleles involved in hybrid incompatibilities are selectively removed, hybrids within a given population should become more similar in fitness to the parentals within that population. Thus, our design ideally would have examined hybrid fitness relative to parents within the Old and New sympatry regions. Unfortunately, the lack of animals limited our ability to perform this design. Nevertheless, previous work (Seidl et al. 2019) is consistent with the possibility that selection in hybridizing populations has removed incompatibilities between the two species. At the level of gene expression, *S. bombifrons* ‐maternal hybrids produced from populations in which hybridization is ongoing were more like pure *S. bombifrons* and *S. multiplicata* than *S. bombifrons* ‐maternal hybrids produced by crossing allopatric pure‐species individuals (a proxy for “first contact” between the species) even though the pure‐species tadpoles remained distinct from one another (i.e., the *Spea* species are not simply collapsing) (Seidl et al. 2019).

A further issue to consider is that the “space for time substitution” design assumes that spatial differences in hybrid fitness reflect changes over time (sensu Wogan and Wang 2018). However, this need not be the case. An alternative, not mutually exclusive, explanation for our results is that fewer incompatibilities might occur between the two *Spea* species in the region of Texas compared to Arizona because of underlying genetic variation in these populations. Population differences in genetic incompatibilities are expected if differential population genetic variation in the gene pools of the two species varies in space or time (Cutter 2012, 2015; Matute et al. 2014; Mandeville et al. 2015). Indeed, *S. multiplicata* potentially consists of two distinct clades (A. Kelly, S. Ryan, B. Stuart, and K. Pfennig, unpublished data; see also Kelly 2020), which might differ in the extent or nature of genetic incompatibilities with *S. bombifrons* . Further work is needed to disentangle these different explanations for our results, especially given the low number of families and populations sampled in our study.

The above limitations and shortcomings of our study underscore the difficulties in using natural populations to experimentally test the hypothesis that selection can evolutionarily ameliorate genetic incompatibilities. Ideally, space for time substitution experiments would involve large numbers of families from many populations, for which much is known about the length of time in sympatry and/or hybridization rates and other potentially confounding variables such as genetic and habitat structure (sensu Lovell et al. 2023). Of additional value in such studies would be prior knowledge of the incompatibilities that impact hybrid fitness (and the extent of allelic variation involved in those incompatibilities) or, alternatively, elements of design that could identify incompatibilities (e.g., obtaining genetic samples from the same individuals for which fitness is measured). Of course, many naturally hybridizing systems might not yet meet these criteria, but as more is known about the dynamics of hybridization in natural systems, greater opportunities will arise to evaluate whether selection can ameliorate genetic incompatibilities. Moreover, experimental studies such as that described here could be complemented with observational and correlational studies (Schilthuizen et al. 2004; Schilthuizen and Lammers 2013) as well as experimental evolution studies (Kalirad et al. 2024). The latter could further clarify predictions that could be more feasibly tested in natural populations.

Despite the difficulty in testing the hypothesis that selection can ameliorate hybrid incompatibilities, doing so is crucial for better understanding not only speciation, but also how gene flow between species impacts the ecological and evolutionary consequences of hybridization and introgression (Cutter 2012, 2015; Coughlan and Matute 2020; Pfennig 2021). Indeed, the possibility that hybrid fitness derived from genetic incompatibilities could vary over space and time has important implications for hybridization's role in adaptation and expansion into new habitats. Selection against hybrids can demarcate zones between species that bound species ranges (Barton and Hewitt 1985). Moreover, genetic incompatibilities can constitute genetic load in populations that depress population fitness and reduce the chances for those populations to persist or serve as a source of dispersers into other populations. Evolutionary reductions of incompatibilities would reduce this genetic load such that adaptive evolution and population persistence are increased as is the likelihood of further expansion by one or the other of the hybridizing species (Barton and Hewitt 1985, 1989; Schilthuizen et al. 2004; Schilthuizen and Lammers 2013) or even species fusion (sensu Seehausen et al. 1997; Behm et al. 2010). This dynamic might be especially important in the early stages of adaptive radiations or between recently diverged species (Gillespie et al. 2020). Given that many populations and species are experiencing decline and that hybridization might impact (or be impacted by) species responses to global change (Taylor et al. 2015; Porretta and Canestrelli 2023), identifying the causes—and consequences—of variation in genetic incompatibilities remains a compelling problem in ecology and evolution.

## Author Contributions

**Sedona Ryan:** conceptualization (supporting), data curation (supporting), formal analysis (supporting), investigation (equal), methodology (supporting), project administration (equal), resources (supporting), supervision (supporting), writing – original draft (lead), writing – review and editing (equal). **Patrick Kelly:** data curation (lead), formal analysis (lead), investigation (equal), methodology (equal), software (lead), writing – review and editing (supporting). **Bryson Loflin:** conceptualization (supporting), data curation (supporting), investigation (supporting), methodology (supporting), project administration (supporting), software (supporting), writing – review and editing (supporting). **Karin S. Pfennig:** conceptualization (lead), data curation (supporting), funding acquisition (lead), methodology (lead), project administration (lead), resources (lead), supervision (equal), writing – original draft (equal), writing – review and editing (lead).

## Conflicts of Interest

The authors declare no conflicts of interest.

## Data Availability

All data and R code are publicly available at UNC Dataverse: https://doi.org/10.15139/S3/JDRBK5.
